# Long Non Coding RNAs (lncRNAs) Are Dysregulated in Malignant Pleural Mesothelioma (MPM)

**DOI:** 10.1371/journal.pone.0070940

**Published:** 2013-08-19

**Authors:** Casey M. Wright, Michaela B. Kirschner, Yuen Yee Cheng, Kenneth J. O'Byrne, Steven G. Gray, Karin Schelch, Mir Alireza Hoda, Sonja Klebe, Brian McCaughan, Nico van Zandwijk, Glen Reid

**Affiliations:** 1 Asbestos Diseases Research Institute, The University of Sydney, Concord New South Wales, Australia; 2 Trinity College, Dublin, Ireland; 3 Flinders Medical Centre, Adelaide, Australia; 4 Department of Cardiothoracic Surgery, Royal Prince Alfred Hospital, and Department of Medical Oncology, Royal North Shore Hospital, Sydney, Australia; 5 Institute of Cancer Research and Division of Thoracic Surgery, Comprehensive Cancer Center, Medical University of Vienna, Vienna, Austria; The Chinese University of Hong Kong, Hong Kong

## Abstract

Malignant Pleural Mesothelioma (MPM) is an aggressive cancer that is often diagnosed at an advanced stage and is characterized by a long latency period (20–40 years between initial exposure and diagnosis) and prior exposure to asbestos. Currently accurate diagnosis of MPM is difficult due to the lack of sensitive biomarkers and despite minor improvements in treatment, median survival rates do not exceed 12 months. Accumulating evidence suggests that aberrant expression of long non-coding RNAs (lncRNAs) play an important functional role in cancer biology. LncRNAs are a class of recently discovered non-protein coding RNAs >200 nucleotides in length with a role in regulating transcription. Here we used NCode long noncoding microarrays to identify differentially expressed lncRNAs potentially involved in MPM pathogenesis. High priority candidate lncRNAs were selected on the basis of statistical (*P*<0.05) and biological significance (>3-fold difference). Expression levels of 9 candidate lncRNAs were technically validated using RT-qPCR, and biologically validated in three independent test sets: (1) 57 archived MPM tissues obtained from extrapleural pneumonectomy patients, (2) 15 cryopreserved MPM and 3 benign pleura, and (3) an extended panel of 10 MPM cell lines. RT-qPCR analysis demonstrated consistent up-regulation of these lncRNAs in independent datasets. ROC curve analysis showed that two candidates were able to separate benign pleura and MPM with high sensitivity and specificity, and were associated with nodal metastases and survival following induction chemotherapy. These results suggest that lncRNAs have potential to serve as biomarkers in MPM.

## Introduction

Malignant Pleural Mesothelioma (MPM) is an aggressive cancer often diagnosed at an advanced stage and characterized by a long latency period (*vis*. 20–60 years) [1]. Currently, the differential diagnosis of MPM is difficult and panels of biomarkers and expert pathologists are often needed to arrive at a definite diagnosis. Median survival rates do not exceed 12–18 months following initial diagnosis and the effect of modestly improved chemotherapy is difficult to recognise [2], [3]. Thus new diagnostic and treatment approaches are urgently needed. The identification of novel diagnostic and prognostic biomarkers is expected to help improve the management of MPM patients.

In order to identify biomarkers and novel therapeutic targets for MPM, a better understanding of MPM biology is required. To this end, much work has focused on mRNA expression profiles and DNA copy number changes in MPM, with microRNA profiling a more recent addition. In contrast, there is little known about the role of long non-coding RNAs (lncRNAs) in MPM. LncRNAs, a class of recently discovered non-protein coding RNAs >200 nucleotides in length, have been found to control every level of gene expression, including post-transcriptional gene regulation via control of protein synthesis, RNA maturation and regulation of chromatin structure, as well as mediation of transcription factor activity [4]. Wang et. al. have suggested the presence of four molecular functions for lncRNAs – as signals, decoys, guides and scaffolds [4]. As signalling molecules, lncRNAs can respond to specific stimuli in a time specific manner, and as such can act as markers of functionally and biologically important events (reviewed in [4]). Secondly, lncRNAs can regulate transcription by acting as independent decoys which negatively regulate effector proteins. As guides, lncRNAs can direct changes in gene expression in either *cis* (neighbouring) or *trans* (distantly located) genes and finally can act as molecular scaffolds. More recently, Gutschner et al suggested eight molecular functions of lncRNAs, these being; regulators of gene expression, sponges which sequester microRNAs preventing inhibition of their target transcripts [5], modulators of protein activity and localisation, as endo-siRNAs that target other RNAs for target degradation, as regulators of alternative splicing, scaffolds and finally as important controllers of chromatin remodelling and histone modifications [6].

Altered expression of lncRNAs has been implicated in a myriad of biological processes including normal tissue development and cancer. Accumulating evidence suggests that their aberrant expression plays important functional roles in cancer biology. For example high expression of metastasis associated lung adenocarcinoma transcript 1 (*MALAT1)* has been associated with metastases and poor outcome in patients with NSCLC [7], [8], and is thought to have an important role in alternative splicing and pre-mRNA processing [9]. Similarly, the Hox transcript antisense intergenic RNA (*HOTAIR*) has been shown to interact with the polycomb repressor complex, and is important in epigenetic control. Up-regulation of this lncRNA has been implicated in breast cancer metastasis [10], tumour recurrence in hepatocellular carcinoma [11] and can regulate *PTEN* methylation in laryngeal squamous cell carcinoma [12]. Taken together, these studies attest to the value of lncRNAs as potential markers and potential targets for therapeutic intervention.

Here we have investigated the role of lncRNAs in MPM biology, by (1) comparing lncRNA expression profiles between MPM cell lines and the normal immortalized human mesothelial cell line (MeT-5A) and selecting candidate lncRNAs found to be differentially expressed, (2) validated expression of these lncRNAs in MPM cell lines and an independent set of MPM tumours and (3) correlated lncRNA expression with nodal metastasis and overall survival.

## Methods

### Ethics Statement

This project was approved by the Human Research Ethics Committees at Concord Repatriation General Hospital (Sydney) and the St. James' Hospital/The Adelaide & Meath Hospital (Dublin). All subjects gave informed written consent at the time of surgery for donation of their tissue for this research.

Unpublished de novo cell lines were created at the Institute of Cancer Research Vienna from patient material obtained during surgical biopsy at the Division of Thoracic Surgery, Comprehensive Cancer Center, Medical University of Vienna. Tissue banking and processing to cell lines was approved by the Ethical Review Board of the Medical University of Vienna and General Hospital Vienna AKH (approval number EK Nr. 904/2009). All patients gave informed written consent for use of their tissue in this research.

### Cell lines and clinical samples

Human mesothelioma cell lines H28, H226, H2052, H2452 and MSTO obtained from the American Type Cell Culture repository (ATCC, Rockville, USA), MM05 (kindly provided by the UQ Thoracic Research Centre, The Prince Charles Hospital, Brisbane [13]) VMC6, VMC6/52A, VMC20, VMC40, VMC23 (kindly provided by Walter Berger, Institute of Cancer Research and Walter Klepetko, Division of Thoracic Surgery, Medical University of Vienna, Austria [14], [15]) were all grown in RPMI with 10% fetal bovine serum (FBS) at 37°C with 5% CO_2_. CLAB and 1988 were kindly provided by Melotti et. al and grown in supplemented medium as previously published [16] (70% MCDB 201,30% DMEM, 2% FBS, 2nM clutamine, 1% penicillin/streptomycin, 10 ng/mL bFGF, 20 ng/ml EGF, 15 μg/ml insulin, 2 μg/ml Heparin). REN cells were obtained from Steven Albeda [17] and grown in Ham's F12 medium. Cells from the normal human mesothelial line MeT-5A were obtained from the ATCC and grown in DMEM with 10% FBS. All medium, FBS and other supplements were obtained from Life Technologies or Sigma. The MPM cell lines consisted of a combination of epithelioid (VMC20, VMC23, VMC6, H226, H28, REN, H2052, H2452, 1988, CLAB) and biphasic (VMC40, MM05, MSTO-211H) subtypes.

The formalin-fixed paraffin embedded (FFPE) tumour tissues used in this study were part of a reported series of extrapleural pneumonectomy patients collected from the Royal Prince Alfred Hospital (RPAH) or Strathfield Private Hospital, Sydney between 1994 and 2009 [18]. Molecular subtyping was performed by formal pathology review (SK). Fresh-frozen mesothelium and benign pleural samples were also collected following debulking surgery at Glenfield Hospital Leicester, and were stored in The Leicestershire Mesothelioma Tissue Bank. All patients gave informed written consent for inclusion of their tissue in this study. Anonymised specimens from 18 patients who had not received preoperative treatment were transferred to St. James' Hospital, Dublin. Subject demographics are provided in Table 1.

**Table 1:** Subject Demographics of the two independent validation cohorts.

	COHORT 1#		COHORT 2*
	*MPM*	*Control*	*FFPE MPM*
**N**	14	3	57
**Median Age (Range)**	60.1 (40–75)	55.2 (39–68)	58 (22–74)
**Sex (N, %)**			
*Male*	11 (78.5)	3 (100)	44 (77.2)
*Female*	3 (21.5)	0 (0)	13 (22.8)
**Histotype (N, %)**			
*Epitheliod*	5 (35.7)	–	43 (75.4)
*Biphasic*	6 (42.9)	–	14 (24.6)
*Sarcomatoid*	3 (21.4)	–	0 (0)

a*RNA extracted from FFPE for measurement of lncRNA expression; # RNA extracted from fresh-frozen tumour tissue, control tissue is benign pleura.

### RNA Isolation

Total RNA was extracted from cell lines using the TRIzol reagent (Life Technologies, Carlsbad, CA), from formalin-fixed paraffin embedded (FFPE) tissues using the QIAGEN FFPE RNeasy kit (Qiagen, Valencia, CA) and from cryopreserved malignant mesothelium and benign pleura using the TRI reagent (MRC, Cincinnati, OH) according to manufacturer's instructions. Prior to nucleic acid isolation, FFPE tissue sections were marked and examined by an anatomical pathologist to guide laser-capture microdissection. RNA quality was assessed using an Agilent 2100 Bioanalyser (Agilent Technologies, Santa Clara, CA) for the microarray samples and quantified using an Implen Nanophotometre (Implen, Munich, Germany). Samples with RNA integrity numbers (RINs) >8.0 were used for microarray analysis. RNA was stored at −80°C until further processing.

### Microarray Data Acquisition and Pre-processing

Microarray profiling experiments were performed according to MIAME guidelines using NCode Human Non-coding RNA microarrays (Life Technologies) representing 17,112 non-coding RNAs and 22,074 mRNA probes. Briefly, 10µg of total RNA was labelled using the Superscript Plus Direct cDNA labelling system (Life Technologies), then hybridised to NCode Noncoding RNA v1.0 microarrays according to manufacturer's instructions. After washing, the slides were stored in liquid N_2_ gas for scanning within 24 hours. Arrays were scanned using an Agilent scanner (Agilent Technologies) at the Ramaciotti Centre for Gene Function Analysis (University of NSW, Sydney, Australia). Data was extracted using Agilent Feature Extraction software FE10.5 and data quality assessed using GeneSpringGX bioinformatics software (V12.0, Agilent Technologies). Probes detected in at least one out of the ten samples were included and baseline transformation was performed to the sample median. Data was normalised to the 75^th^ percentile. All cell line experiments were performed in duplicate. All expression data has been deposited in the National Centre for Biotechnology Information (NCBI) Gene Expression Omnibus under accession GSE48174.

### Quantitative reverse-transcription Polymerase Chain Reaction (RT-qPCR)

Long noncoding RNA expression levels from microarray analysis were validated using RT-qPCR in the five cell lines assayed using NCode microarrays and in a panel of primary and ATCC-sourced MPM cell lines. In addition, expression levels were also validated in cryopreserved pleural and mesothelioma tissue and FFPE MPM tissues. Primers were designed using the Universal Probe Library (UPL) algorithm provided by Roche (http://www.roche-applied-science.com/sis/rtpcr/upl/index.jsp?id=UP030000; See Table 2 for primer sequences). Where possible, SYBR green primers were designed as close as possible to the microarray probe to ensure microarray data was reproducible. In some instances, primers could not be designed to the NCBI target lncRNA sequence using default settings with UPL and were not pursued for further analysis. Total RNA (250 ng for cell lines, 50 ng for FFPE and fresh-frozen tissue) was reverse transcribed to cDNA using the AffinityScript qPCR cDNA synthesis kit (Agilent Technologies) using a combination of random hexamers (100 ng/µl) and Oligo (dT) primers (100 ng/µl) in a 10 µl reaction. After reverse transcription, cDNA was diluted 1:5 with 2 µl of this product used as template in RT-qPCR using 180nM of forward and reverse primer. All reactions were run in triplicate on a Stratagene Mx3000P real-time machine (Agilent Technologies) using Brilliant II SYBR Green (Agilent Technologies). No template and no-RT samples were included as negative controls. All reactions had an initial enzyme inactivation step at 95°C for 10 minutes followed by 40 cycles of 95°C for 15 seconds and 55°C for 30 seconds. 18S ribosomal RNA was used as the reference for qPCR data normalisation. Relative expression levels were calculated using the 2-ΔΔCq method described by Pfaffl [19] with MeT-5A designated a value of 1 (all fold changes were calculated relative to this value). Genes were deemed technically replicated if the direction of expression was consistent with microarray data and the magnitude of change was greater than 3-fold.

**Table 2:** SYBR Green Primer Sequences used for microarray validation of the top 23 lncRNAs identified as being differentially expressed between MeT-5A mesothelial cells and MPM cell lines.

NCode Probe ID	Target ID	Chromosomal Position (Hg18)	Forward Primer	Reverse Primer
IVGNh23506	EF177379	chr11:64949929-64949989	actgtcgttgggatttagagtgt	cacaacagcatacccgagac
IVGNh32740	AK130275	chr2:113714740-113714800	ggcagggttaagggaaaaag	cagctctggcagaaccactaa
IVGNh25923	AF268386	chr1:161022613-161022673	acaggacacccgaatcaaaa	ttcaaataggctgggtatgagg
IVGNh35454	NR_003584	chr4:119420005-119420257	acgatggatgatggaaacataa	tccccaactacgataagtcca
IVGNh12568	AK129685	chr19:13810366-13810426	tttgtgaaacgggcagtct	cccagcagtgcaacattaaa
IVGNh16972	BC031859	chr16:4551527-4551587	tgctttttagaagccttcatcc	ggatgggcagataccagga
IVGNh30904	AX746718	chr17:55867220-55867280	aatgcaatagaaaaagaaaaactcg	gggacaaccgaagaaagttg
IVGNh38714	AK054908	chr9:138740661-138741350	ctggacgtctgctcactgg	agtccatcacaggcgaagtc
IVGNh37463	BX648695	chr7:124447674-124508032	agctttgtctcgtggagtctg	tgtgtaacaagttgcattaaaatcct
IVGNh13194	AK130977	chr20:34612610-34612670	tcagtcccaaacacattctcac	atgggtggccagactgag
IVGNh23633	G30815	chr7:30435183-30435243	gcctgattccatattctgtgc	cccaatgacggtagatgagg
IVGNh01065	AF056184	chr4:49207176-49207237	tacgctgcacaactggaagt	ccgttgaaggactcaaacaga
IVGNh11100	AK126075	chr18:41272277-41281247	caatcacttgagtgaacttgagagt	gtgctggacacccagtcag
IVGNh33458	AX746738	chr20:30702313-30702509	cctttacaaaaactggaatgctg	ctcgctgagcctttgagg
IVGNh21169	BX648304	chr9:130706223-130706283	caagttttaaatgcctgtgtcaa	agaaaggtggcccatcatc
IVGNh12928	AK130470	chr9:14215472-14215532	gcacaagctcaatcattgct	tgcagtgagcccagatcc
IVGNh11385	AK126582	chr19:63770592-63770652	cagctctgagcaggtagatgtg	gagtggggttcccctatgtc
IVGNh08061	AK094765	chr21:33318726-33319167	agtattttcagtgtcgtctttgtga	cggggaagagattagggact
IVGNh24779	U17623	chr21:38693604-38693665	catgggggattgagttcct	ggaaccacttctagcaatacagg
IVGNh29716	BC121819	chr15:72560296-72560492	ctcaagtgccgccaaact	agccatctgtgtccatagca
IVGNh17420	BC035363	chr8:144866936-144867206	ccataactgcactgccacac	caaggaggcgtgtcctca
IVGNh17438	BC035497	chr1:45543009-45543069	tgcagtctctctctttgtgacc	gctctgacctggcactctgt
IVGNh09032	AK097452	chr15:78513886-78515514	ctcgtaagatgttgagacttcacc	tgacaaagccagagaagagaga

### Identification of an lncRNA prediction panel capable of distinguishing normal mesothelium and MPM

For the nine candidate lncRNAs identified as being consistently differentially expressed by RT-qPCR, class prediction analyses were performed to determine if this panel of lncRNAs could accurately predict normal/tumour class. All class prediction analyses were performed in BRB ArrayTools V4.1.0β (developed by Dr Richard Simon and Amy Peng Lam http://linus.nci.hih.gov/,brb/tool.htm) using a random variance model developed on a significance level of *P* = 0.05 [20]. Six different prediction models were used for classifier development: (1) Compound covariate, (2) Diagonal linear discriminant analysis, (3) K-nearest neighbour, (4) Nearest centroid, (5) Support vector machines, and (6) Bayesian compound covariate model. The misclassification error rate was predicted using a leave-one out cross-validation (LOOCV) procedure. In the first instance this predictor was tested using the microarray data obtained from the NCode microarrays, and then it was validated using RT-qPCR data obtained from mesothelioma cell lines and fresh-frozen tissues to confirm overall sensitivity and specificity.

### Gene ontology and Pathway analysis

Over-represented gene ontologies and pathways were determined using the gene functional annotation tool, forming part of the DAVID annotation software package (http://david.abcc.ncifcrf.gov/home.jsp) as described previously [21]. Coding genes identified as being highly correlated with lncRNA expression were compared with all genes contained in the DAVID database. Pathways and gene ontologies with Fisher's exact probability P-values less than *P* = 0.05 were considered significantly enriched.

### Statistical analyses

Differential microarray expression analysis was performed using GeneSpring v12.0 using unpaired t-tests. Candidate genes were selected on the basis of (a) statistical significance of expression (*P*<0.05*)* and (b) magnitude of expression change (>3-fold difference). For RT-qPCR, gene expression data was analysed using the Pfaffl method for relative quantitation, and normalised to 18S. Group comparisons, correlations and associations were performed using SPSS statistical software and two tailed Mann-Whitney U t-tests. A *P*-value less than 0.05 was considered statistically significant. Kaplan-Meier curves and log rank tests were used to assess survival differences between groups with multivariate analyses performed to assess the degree of interaction between different factors. All experiments were performed in triplicate to ensure reproducibility of results.

## Results

### LncRNA expression profiles in Malignant Pleural Mesothelioma (MPM) cell lines

**Figure 1:**
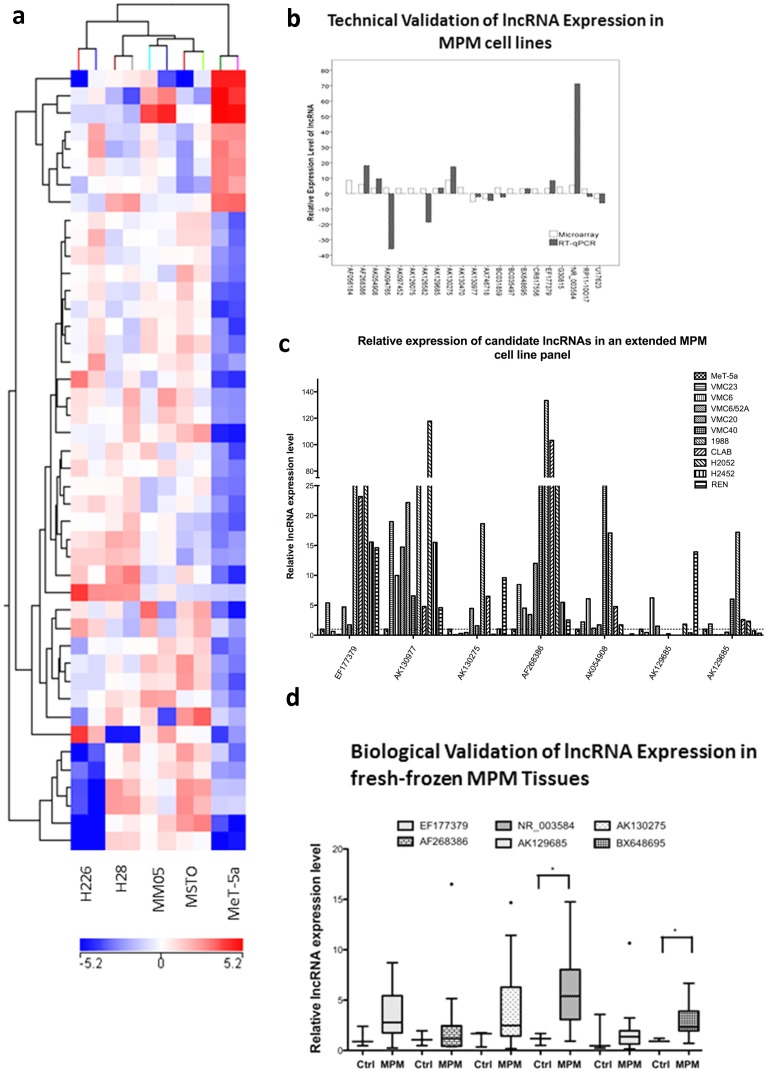
RT-qPCR validation reveals significant lncRNA expression differences in MPM cell lines and fresh-frozen tissue. Levels of lncRNA expression were normalised to 18S and relative expression levels compared to the average level in the control samples for MPM tissues and MeT-5A for cell lines using the 2^−ΔΔCq^ method. (a) Unsupervised cluster analysis of the top 44 lncRNAs found to be differentially expressed between MeT-5A and MPM (H226, H28, MSTO, MM05) cell lines using NCode Long Noncoding RNA microarrays. All cell lines were profiled in duplicate. Red  =  regions over-expressed, Blue  =  regions under-expressed. (b) Nine candidate lncRNAs were technically validated in MPM cell lines using RT-qPCR. For RT-qPCR, lncRNA expression levels were normalised to 18S and are expressed relative to MeT-5A. (c) NR_003548 and BX648695 were significantly elevated in MPM tissues compared to benign pleura. Turkey box plots have median values represented by the line within the boxes, and the 25^th^ and 75^th^ percentiles represented by the upper and lower lines of the box. (d) 7 candidate lncRNAs were biologically validated in an extended panel of 10MPM cell lines. All candidates demonstrated consistent up-regulation of expression. MPM – Malignant Pleural Mesothelioma, lncRNA – long noncoding RNA, Ctrl – Benign Pleura, * statistically significant at P<0.05 (two-tailed t-test).

In order to identify new markers for MPM, we used microarrays to profile lncRNA content in 5 cell lines (4 MPM, and 1 normal mesothelial cell line). Class comparison analyses were performed to identify highly differentially expressed lncRNAs in MPM. In total, this approach identified 44 lncRNAs highly differentially expressed in MPM cells compared to MeT-5A. Unsupervised hierarchical cluster analysis demonstrated variations in the expression profiles of MeT-5A and the four MPM cell lines (Figure 1a) suggesting the possibility that an lncRNA signature could exist. After checking the annotations of candidates, eleven candidates were reclassified as mRNA transcripts and were excluded, leaving 33 candidate lncRNAs for potential follow-up (Table 3).

**Table 3:** List of significantly differentially expressed long noncoding RNAs identified from NCode Microarray analysis using statistical (*P*<0.05) and biological criteria (>3-fold).

MICROARRAY ANALYSIS						TECHNICAL VALIDATION – CELL LINES1			BIOLOGICAL VALIDATION SET 1- DUBLIN TUMOUR SET		
ProbeName	Target ID	Subclassification	P-value	Direction of Expression	Absolute Fold Change	Absolute Fold-change #	Direction of Expression	P-value	Absolute Fold-change #	Direction of Expression	P-value
IVGNh01065	AF056184	Intergenic	0.036	up	8.517	ND	ND	ND	-	-	-
IVGNh25923	AF268386	Intergenic	0.036	up	5.917	18.20	up		2.17	up	
IVGNh38714	AK054908	3′UTR, Bidirectional	0.022	up	3.470	9.66	up		ND	ND	
IVGNh08061	AK094765	Intergenic	0.022	up	3.814	36.00	down		-	-	-
IVGNh09032	AK097452	Cis – antisense	0.022	up	3.302	ND	ND	ND	-	-	-
IVGNh11100	AK126075	Intergenic	0.036	up	3.381	ND	ND	ND	-	-	-
IVGNh11385	AK126582	Intergenic	0.022	up	3.222	18.70	down		-	-	-
IVGNh12568	AK129685	Intergenic	0.022	up	3.299	3.70	up		ND	ND	
IVGNh32740	AK130275	Bidirectional, cis-antisense	0.036	up	8.704	17.50	up		3.3	up	
IVGNh12928	AK130470	Intergenic	0.022	up	4.040	ND	ND	ND	-	-	-
IVGNh13194	AK130977	Intergenic	0.048	down	5.207	1.60	down				
IVGNh30904	AX746718	Intergenic	0.022	down	3.373	4.60	down		ND	ND	
IVGNh16127	BC017840	Intergenic	0.022	up	7.729	Record not found			-	-	-
IVGNh16972	BC031859	Cis – antisense	0.022	up	3.831	2.41	down		-	-	-
IVGNh17420	BC035363	Bidirectional	0.022	down	3.222				-	-	-
IVGNh17438	BC035497	Intergenic	0.022	up	3.126	ND	ND	ND	-	-	-
IVGNh29716	BC121819	Intergenic, Bidirectional	0.022	up	3.103	2.00	down		-	-	-
IVGNh21169	BX648304	Intergenic	0.022	up	3.043				-	-	-
IVGNh37463	BX648695	Intergenic, Bidirectional	0.036	up	3.125	3.28	up		3.2	up	
IVGNh33458	C20orf203	Intergenic,	0.022	up	3.380						
IVGNh30174	CR617556	Intergenic	0.022	down	3.072	Record Removed from NCBI	-	-	-	-	-
IVGNh23506	EF177379	3′UTR	0.022	up	3.408	8.45	up		2.8	up	
IVGNh23633	G30815	Intergenic	0.022	up	4.350	ND	ND	ND	-	-	-
IVGNh38595	NR_002449 (SNORA65)	Intronic, Promoter associated	0.022	up	5.453	NP	-	-	-	-	-
IVGNh25978	NR_002746	Intergenic, Birectional	0.022	up	3.589	NP	-	-	-	-	-
IVGNh37000	NR_003075.1	Intergenic	0.048	up	3.459	NP	-	-	-	-	-
IVGNh35454	NR_003584	Intergenic	0.022	up	5.322	71.30	up		5	up	
IVGNh29372	SNORD116-12	Intergenic	0.048	up	3.212	NP	-	-	-	-	-
IVGNh29374	SNORD116-23	Intergenic	0.036	up	3.135	NP	-	-	-	-	-
IVGNh24779	U17623	Intergenic	0.022	up	3.291	6.15	down	-	-	-	-
IVGNh24857	U52835	Intergenic	0.022	up	3.544	NP	-	-	-	-	-
IVGNh33847	uc002zjh	Intergenic	0.048	down	5.422	NP	-	-	-	-	-

#NP – no primers could be designed, ND – Not detected; – not followed up biologically due to lack of technical replication or original primers not designed, Fold changes for cell lines are expressed relative to MeT-5A.

To identify lncRNAs for subsequent experimental analysis, we further refined our list of lncRNAs by examining their genomic context in an attempt to better understand the possible functional role these candidates may have. Previous studies have shown that many lncRNAs originate from complex transcriptional loci, and potentially have functional relationships with their nearby coding genes [22]. The lncRNA-containing loci were grouped into one of six categories as described by Dinger et. al.: (1) cis-antisense, (2) intronic, (3) bidirectional, (4) present in the 3′UTR, (5) promoter-associated or (6) intergenic [23]. Of the 33 lncRNAs most differentially expressed; 22 lncRNAs were intergenic, 3 were intergenic/bidirectional, 2 cis-antisense, 2 in the 3′UTR, 1 bidirectional/cis-antisense, 1 bidirectional only, 1 bidirectional/3′UTR, and 1 that was intronic/promoter associated (Table 3).

### mRNA expression profiles differ between MPM and MeT-5A cell lines

In addition to the lncRNA content we also profiled mRNA content using the NCode microarrays. Class comparison analyses were performed as described for lncRNAs, with high priority candidates selected on the basis of statistical *(P<*0.05) and biological significance (>3-fold change). In total this approach identified 305 mRNA probes representing 249 genes differentially expressed between MeT-5A and MPM cell lines: 151 probes were down-regulated and 155 probes were up-regulated in MPM compared to MeT-5A. Interrogation of the Oncomine database identified 28 of these 249 genes in the top 10% under- or overexpressed genes in MPM compared to normal pleura [24], [25] Some of these genes included *ANXA4*, *GALTN7, MET RBMS1, TM4SF1,* and *UCHLI.* A list of these genes is provided in Table S1 in File S1. Furthermore, Crispi et. al. identified aberrant expression of *CDKN2A, TK1*, *MYC* and *STMN1* [26] Further confirmation in MPM cell lines also confirmed dysregulation of *TSM4SF1, STMN1, IFI16, CDKN3* and *ARHGDIG* [27]. Gene ontology analysis of the top 305 mRNA probes revealed over enrichment of biological processes including cell cycle (*P = *0.0000048), apoptosis (*P = *0.00022), cell death (*P* = 0.00029), cytoskeleton organisation (*P = *0.00043), regulation of kinase activity (*P = *0.012), positive regulation of caspase activity (*P = *0.039) and positive regulation of the IκB kinase/NFκB cascade (*P = *0.034). Table S2 in File S1 provides a complete list of over-enriched gene ontologies.

### Technical Validation of Candidate lncRNAs

Next, lncRNA expression was confirmed using RT-qPCR in the microarray training set consisting of 4 MPM cell lines and the normal mesothelial line MeT-5A. Primers were successfully designed for 22 (represented by 23 microarray probes)/33 lncRNAs (33 probes) demonstrating significant differential expression in MPM cell lines (See Table 2 for primer sequences). For the remaining 11 candidates, primers could not be successfully designed using UPL default settings due to the smaller fragment size. All targets were normalised to 18S. Of the 22 lncRNAs (represented by 23 probes) assessed, 8 were not detected in any of the cell lines, 5 did not validate in the correct direction (i.e. direction of expression was different for microarray and qPCR data) and 9 lncRNAs were validated. A gene was considered technically validated if the direction of expression change between MeT-5A and MPM cell lines was consistent for both microarray and RT-qPCR and the magnitude of difference was greater than 3-fold.

After normalisation to 18S, the levels for the 14 detectable lncRNA candidates varied from 1.6-fold to 71.3 fold (Table 3; Figure 1b). AK130977 and AX746718 were both found to be down-regulated via both microarray and RT-qPCR (AK130977 Microarray (MA)  = −5.207, RT-qPCR  = −1.6; AX746718 MA  = −3.37, RT-qPCR  = −4.6), with AK130977 demonstrating fairly small changes using RT-qPCR. Similarly, BX648695, AK129685, EF177379, AK054908, AK130275, AF268386 and NR_003584 all demonstrated consistent up-regulation using both microarrays and RT-qPCR.

### Analysis of lncRNAs in MPM tumours

lncRNA expression of the nine technically validated targets was validated in an extended panel of 10 MPM cell lines and expression calculated relative to the immortalised mesothelial line MeT-5A. The majority of candidates demonstrated a >3-fold up-regulation in MPM cell lines compared to MeT-5A (Figure 1c). For EF177379, MPM cell lines demonstrated a 14.6 fold up-regulation, with 6/10 cell lines demonstrating >5-fold difference in expression. High levels were also observed for AK130977 (average 25-fold upregulation; 10/10 cell lines >4-fold differences in expression) and AF268386 (38-fold upregulation; 7/10 cell lines >5-fold differences).The remaining candidates AK130275, AK054908, AK129685 and NR_003584 also demonstrated moderate levels of up-regulation (3–6 fold) although not all cell lines demonstrated overexpression.

**Figure 2:**
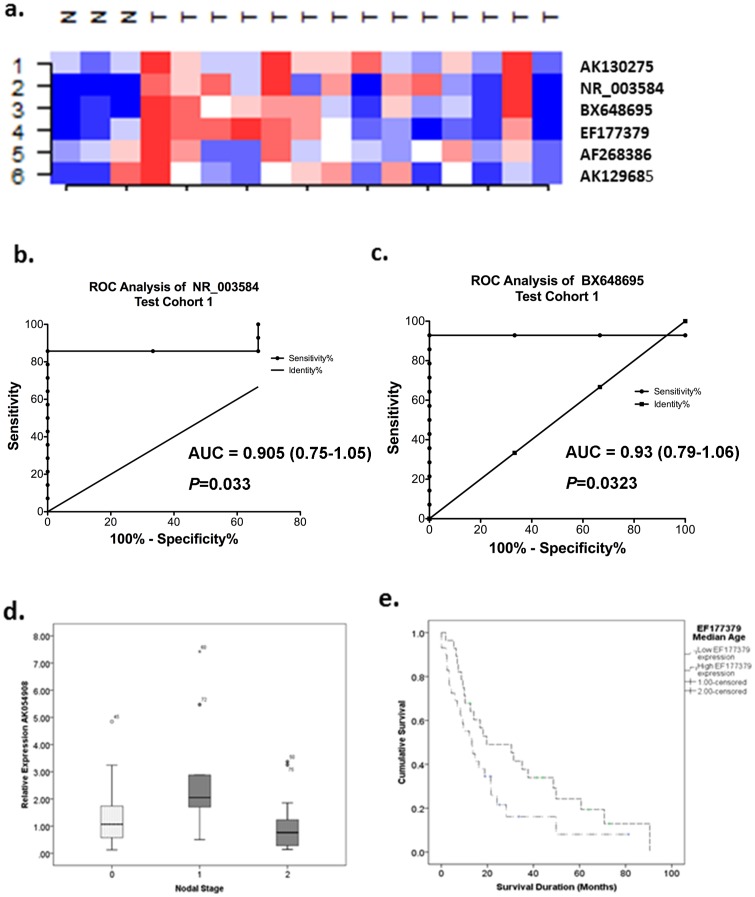
Class prediction profiling using the six biologically validated lncRNAs demonstrates high sensitivity in predicting MPM class in the NCode Microarray data (data not shown) and cryopreserved MPM and benign pleural tissue. (a) Supervised cluster analysis shows that this predictor demonstrated overexpression (red areas) in MPM tumours compared to benign pleura. Roc curve analysis shows that (b) NR_003584 and (c) BX648695 can clearly separate benign tumour and MPM tissue with a high degree of accuracy. (d) AK054908 lncRNA expression is associated with hilar lymph node metastasis. (e) Higher EF177379 expression is associated with longer survival.

The expression of the nine targets was then validated in 14 MPM and 3 benign pleura cryopreserved following surgical resection. All three pleural samples showed no malignancy: Case 1 had benign hyaline plaques with chronic inflammation and reactive changes (pleural plaques), Case 2 (pneumothorax) had evidence of chronic inflammatory infiltrate and mesothelial proliferation and Case 3 (empyema) had acute and chronic inflammation with fibrosis. Six lncRNAs were detectable. Supervised cluster analysis showed that the expression patterns for these lncRNAs were different between normal pleura and MPM tissues (Figure 2a). AK130275, AK129685, EF177379 and AF268386 all demonstrated >-2-fold expression differences in MPM compared to benign pleura without reaching significance (AK130275 – 3.3 fold, MWU *P* = 0.345; AK129685 – 3.2 fold, MWU *P* = 0.659; EF177379 – 2.8 fold, MWU *P* = 0.186; AF268386 – 2.17 fold, MWU *P* = 0.950). The remaining two lncRNAs, BX648695 and NR_003584 both demonstrated significant expression differences in MPM compared to benign pleura (BX648695 – 2.95 fold, MWU *P* = 0.028; NR_003584- 5-fold, MWU *P* = 0.038). Receiver operating characteristic (ROC) curve analysis showed that BX648695 could discriminate benign pleura and MPM with an accuracy of 93%, sensitivity 78.6% and specificity 100% (AUC 0.93, 95% confidence interval 0.793–1.064, *P = *0.023; Figure 2b). Similarly NR_003584 could discriminate benign pleura and MPM with an accuracy of 90.5%, sensitivity 78.5% and specificity 100% (AUC 0.905, 95% confidence interval 0.752–1.057, *P = *0.033; Figure 2a). While the remaining lncRNAs did not demonstrate significant differences between benign pleura and MPM, it does not exclude these targets as potential markers given the small test cohort used (Figure 1d).

### Identification of lncRNA expression profiles to classify normal mesothelium and MPM

To determine whether lncRNA expression profiles are capable of distinguishing normal mesothelium and MPM cells, we performed class prediction analyses using the six lncRNAs validated/detected in both (1) the original microarray dataset and (2) RT-qPCR data from the MPM and benign pleural samples. These six lncRNAs were able to correctly classify MPM and MeT-5A at *P*<0.05, using the NCode microarray data. The worst performing model was the 3-neighbour algorithm which correctly classified 80% of samples. While able to correctly classify all MPM cell lines, it was unable to correctly predict MeT-5A as normal. In contrast the remaining models were able to correctly classify 100% of samples with 100% sensitivity and specificity. The best performing models were the compound covariate, diagonal linear discriminant analysis and nearest centroid classifiers which were all capable of clearly distinguishing MPM and MeT-5A at *P* = 0.056 *(*based on 1000 permutations of the class label).

Validation of this signature in the RT-qPCR data from the 3 benign pleura and 14 MPM tissues showed that the nearest centroid and compound covariant predictors (CCP) were the best performing models correctly classifying 76% of samples with a sensitivity of 71.4%, specificity 100%, PPV 100% and NPV 43% (CCP *P* = 0.179, Nearest centroid *P* = 0.169 based on 1000 permutations of the class label). In contrast, the remaining models correctly classified tumours 59–71% of the time with variable sensitivity and specificity. Overall, this lncRNA signature had good sensitivity for detecting MPM however assessment in a larger cohort is required.

### LncRNA expression is associated with nodal stage and prior treatment with induction chemotherapy

Of the nine technically validated lncRNAs, four candidates were detected in an independent cohort of fifty-seven archived FFPE tissues and were correlated with clinical covariates including age, survival time, T stage, nodal involvement, and previous induction chemotherapy. We observed no significant relationship between lncRNA expression and histological subtype.

Higher expression of AK054908 was significantly associated with metastasis in the ipsilateral bronchopulmonary/hilar lymph nodes (N1 status; AK054908 *P* = 0.005; Figure 2d) with a trend for higher expression also observed in AF268386 (*P* = 0.071). Significantly lower levels of AK130275 and AF268386 were observed in patients receiving induction chemotherapy (AK130275 induction chemotherapy  = 0.763 vs. no chemotherapy 1.578, *P* = 0.002; AF268386 induction chemotherapy  = 0.956 vs. no chemotherapy 1.685, *P* = 0.033). No significant differences were observed between candidate lncRNA expression levels and tumour stage, histology or sex.

Stratification of lncRNA expression levels into high and low expression groups based on median expression values revealed no significant survival differences between groups. However, cases with higher EF177379 (*NEAT1*) expression demonstrated a trend towards improved survival compared to cases with expression levels below the median (low expression 13.3 months (95% CI 5.9–20.6 months) versus high expression group 19.3 months (95% CI 1.5–37.9 months) *P* = 0.065; Figure 2e)). When stratified by treatment with/without induction chemotherapy, patients with higher EF177379 levels had better overall survival when they did not receive induction chemotherapy (Log-Rank *P = *0.019, median survival low expression  = 9.5 month versus high expression 19.7 months). Lower EF177379 levels were present following treatment with induction chemotherapy.

### Genes co-expressed with lncRNAs are associated with cellular and metabolic processes

Next, we constructed a coding-noncoding gene network to investigate potential relationships between the top 6 lncRNAs and 305 mRNAs found to be differentially expressed between MPM and MeT-5A cell lines. Pearson correlations were used to compare lncRNA and mRNA expression levels from the microarray. Genes with correlation co-efficients >0.6 and *P*-values <0.05 were taken for additional pathway analysis to identify ontologies/pathways associated with lncRNA dysregulation (See Table S3 in File S1 for correlation coefficients, and Table S4 in File S1 for gene ontologies).

Assessment of mRNAs co-expressed with AK130275 found over-enrichment of genes involved in cell death *(P* = 0.049) and epithelium development *(P* = 0.026). In addition, >50% of mRNAs were associated with phosphoproteins *(P* = 0.003; 56% genes) and alternative splicing *(P* = 0.024; 52%). For BX648695, 22 unique genes were co-expressed and were associated with muscle system processes *(P* = 0.047), calcium ion binding *(P* = 0.024), metal ion binding *(P* = 0.019) and phosphoproteins *(P* = 0.016). AF268386 co-expressed mRNAs were significantly associated with actin-filament based processes *(P* = 0.049), glycolipid metabolism *(P* = 0.045), cell proliferation *(P* = 0.025), cell cycle (*P* = 0.025), ganglioside metabolic processes (*P* = 0.012), and Rho GTPase binding (*P* = 0.051) with some of these processes demonstrating a greater than 10-fold over-enrichment. EF177379 (*NEAT1*) was found to be co-expressed with 139 mRNAs involved in processes including cell cycle (*P*<.001), apoptosis (*P*<0.001), cell death (*P* = 0.01), regulation of cyclin-dependent protein kinases (*P* = 0.046), cell cycle arrest (*P* = 0.028), M phase (*P* = 0.018) and cytoskeleton reorganisation (*P* = 0.021). Several of these genes were also associated with non-small cell lung cancer (NSCLC; KEGG pathway; *P* = 0.010).

Enrichment of cell death (*P*<0.001), apoptosis (*P*<0.001), regulation of apoptosis (*P* = 0.01), regulation of protein kinases (*P* = 0.04) and cell cycle (*P* = 0.046) related processes were associated with AK129685 co-expression. Finally, mRNAs co-expressed with NR_003584 were associated with enrichment of cellular processes including cell cycle (*P*<0.001), apoptosis (*P = *0.002), actin cytoskeleton organisation (*P*<0.001), mitotic cell cycle (*P*<0.001), regulation of protein kinase activity (*P* = 0.004), regulation of transferase activity (*P* = 0.006), cell division (*P* = 0.021), regulation of phosphorylation (*P* = 0.021), regulation of phosphate metabolism (*P* = 0.026), and cell morphogenesis (*P* = 0.046).

## Discussion

Since the realisation that non-coding regions of the genome are functional and not “junk” as previously thought, there have been numerous studies linking changes in long noncoding RNA (lncRNA) expression to cancer prompting an increasing interest in the use of lncRNAs as potential disease biomarkers [7], [28]–[30]. There is however, still a lack of knowledge surrounding the functional implications of lncRNA dysregulation and the precise role they play in carcinogenesis. To our knowledge this is the first systematic study of lncRNA expression profiles in MPM. We have found that lncRNA expression profiles can distinguish malignant mesothelium and benign pleura, and that some lncRNAs are associated with nodal metastasis and long term survival.

To investigate lncRNA expression patterns in MPM, we used whole-genome lncRNA expression microarrays to profile four MPM cell lines, and the immortalised human mesothelial cell line MeT-5A. As MPM is a rare tumour, it is often difficult to obtain enough tissue for research purposes, thus we theorised that if candidate lncRNAs identified from cell line data could be validated in MPM tissue, then these candidates were likely to have a biological role in MPM. While recognising that MPM cell lines often demonstrate additional changes that reflect their adjustment to *in-vitro* conditions, the fact that the candidates were validated in tumour tissues provides evidence for a biological role in MPM. We ranked candidate lncRNAs on the basis of statistical significance (P<0.05) and fold change (>3-fold), and validated expression using RT-qPCR. We identified a panel of nine lncRNAs, six which were biologically validated in cell lines and MPM tissues (AK130275, AK129685, EF177379, BX648695, NR_003584 and AF268386) and seemed to be diagnostic. In FFPE tissues from MPM patients four lncRNAs were detectable. Two of these lncRNAs were associated with nodal metastases and overall survival. Gene ontology and pathway analyses were performed on co-expressed mRNA-lncRNA networks to investigate possible biological functions of these lncRNAs.

The lncRNA EF177379 (*NEAT1)*, is located on 11q13.1, a region reported to be amplified in MPM. It has been implicated in mRNA transport regulation, is associated with the 3′ untranslated region (3′UTR) and is an important structural component of paraspeckles [31]. *NEAT1* has been shown to control the retention of Alu-containing mRNAs in the nucleus with growing evidence emerging to suggest that gene expression can be regulated by retained mature mRNAs [32]. These retained mRNAs may be inefficiently transported out of the nucleus in paraspeckles. Considering that cancer cells have altered mechanisms for nucleoplasmic mRNA transport [33], it is possible that *NEAT1* may play a role in regulating this pathway in cancer. It is also tempting to speculate that its expression may also be regulated by copy number gains.

AK130275 is a bidirectional lncRNA located antisense to *PAX8,* a transcription factor expressed at high levels in the thyroid, that has an essential role in cell proliferation [34]. Bidirectional lncRNAs have been shown to exhibit similar expression patterns to their nearby protein-coding genes and are possibly involved in similar gene regulatory mechanisms. Immunohistochemical studies have shown that *PAX8* expression can help to distinguish ovarian cancer and pleural/ peritoneal mesotheliomas [35], with mesotheliomas demonstrating weak to negative staining of *PAX8.* A recent meta-analysis of 15 ovarian cancer studies, reported that prior exposure to asbestos is associated with an elevated risk of ovarian cancer [36] with ovarian cancers capable of metastasising to the pleura [37]. Considering its close proximity to *PAX8*, it is possible that AK130275 may regulate expression of *PAX8* by stabilizing the corresponding mRNA or exert its own functional effects. Such a regulatory mechanism has been described for sense-antisense pairs which show a partial overlap either in 5′UTR [38] or 3′UTR regions [39]. Thus AK130275 could be potentially useful for the differential diagnosis of primary and metastatic pleural mesothelioma.

AK054908, also known as the small nucleolar RNA host gene 7 (*SNHG7*), has been described as a 3′UTR/bidirectional lncRNA and is thought to encode the smaller snoRNAs, SNORA43 and SNORA17. SnoRNAs guide RNAs for post-translational modification, in the process modifying ribosomal RNA and are often located in introns/exons of coding and noncoding transcripts. Other cancer pathways including the mTOR pathway have also been implicated in ribosome biogenesis and have been previously shown to be important for promoting transcription of ribosomal RNAs. Ribosome biogenesis is critical for protein synthesis during cell growth and proliferation [40]. In mesothelioma, inhibition of this pathway has been shown to inhibit cell invasion, motility and spreading [40]. Whether overexpression of SNHG7 contributes to altered ribosomal biogenesis and ultimately cell growth and proliferation remains to be determined, but it is interesting to note that we found this gene to be associated with nodal metastasis, suggesting it may be potentially useful as a prognostic marker.

Finally, AF268386 is a long intergenic lncRNA (or long intergenic noncoding RNA – lincRNA). Intergenic lncRNAs have been implicated in a variety of biological processes including cell cycle regulation and immune surveillance, and often work with chromatin modifying complexes to influence gene regulation. Epigenetic changes including DNA methylation have been well reported in MPM with hypermethylation of E-Cadherin (*ECAD*; 71.4%), fragile histidine triad (*FHIT*; 78%), the secreted frizzled related protein family (*SFRP*s), *RASSF1A* (19.5%), *DAPK* (20%) and *RARB* (55.8%) all being implicated in MPM [41], [42]. The fact that these changes occur relatively frequently suggests that regulatory mechanisms controlling these processes become aberrant during the carcinogenic process, with lncRNAs being one potential source of epigenetic disruption.

Next, to better understand the functional impact of these lncRNAs, we applied a pathways approach to study the relationship between differentially expressed lncRNAs and mRNAs. We found that our candidate lncRNAs were co-expressed with mRNAs involved in a variety of cell processes including cell death, cell proliferation, apoptosis, glycolipid metabolism, Rho-GTPase signalling, cell cycle, DNA replication, recombination and repair, actin cytoskeleton reorganisation and regulation of the protein kinase cascade. All of these pathways are key processes involved in cancer progression suggesting that these lncRNAs may play critical roles in cell cycle regulation. Several of the mRNAs identified as being differentially expressed in our study have also been identified in previous MPM gene expression studies, strengthening the validity and quality of our data, and highlighting the likely biological role of these candidates [25], [27], [43]. Integration of lncRNA expression profiles with mRNA and microRNAs profiles is likely to provide insights into the precise roles these lncRNAs are playing. It is possible these lncRNAs act in negative or positive feedback loops as oncogenes or tumour suppressor genes.

There is also evidence to suggest that ncRNAs may interact with microRNAs to form extensive regulatory networks [5]. Termed competing endogenous RNAs (ceRNA), these ceRNAs can protect target RNAs from repression by sequestering microRNAs [5]. In hepatocellular carcinoma (HCC), the maternally expressed gene 3 (*MEG3*) was found to be hypermethylated and down-regulated by 210-fold in HCC compared to non-malignant hepatocytes. Investigation of microRNA dependent regulation by miR-29a, showed that its overexpression was associated with a methylation dependent and tissue specific increase in MEG3 expression [44]. In muscle differentiation, linc-MD1 contains recognition sites for miR-133 and miR-135 with its depletion found to reduce levels of two predicted targets of miR-133 and miR-135, *MAML1* and *MEF2C* [45]. This suggests that linc-MD1 may act as a decoy for miR-133 and miR-135. In addition, *PTENP1* a lncRNA antisense to *PTEN*, shares a similar 3′UTR transcript to *PTEN* [46]. Mutations in this region have been shown to disrupt microRNA binding, negating the protective effect of the lncRNA transcript, [46]. Taken together these studies highlight the interdependence observed between lncRNAs and microRNAs, and suggest that lncRNAs and microRNAs work together in complex regulatory networks to activate or suppress gene expression.

The recent implementation of large scale next-generation sequencing has provided vast insights into gene regulation, with accumulating evidence suggesting that lncRNAs have important functional roles in cancer development. LncRNAs may therefore be useful diagnostic and prognostic biomarkers especially given their higher tissue specificity. For example the non-coding RNA prostate cancer antigen 3 (*PCA3*) has been shown to improve the diagnosis of prostate cancer in a more sensitive and specific manner than the widely used *PSA* (prostate-specific antigen), and is detectable in urine from patients with prostate cancer [47] while, the highly upregulated in liver cancer (HULC) lncRNA has been detected in blood from patients with hepatocellular carcinoma (HCC) [48]. Many lncRNAS have also been implicated in the control of metastasis, apoptosis and drug resistance including *MALAT1* which has been shown to be prognostic in early-stage lung adenocarcinoma and *HOTAIR* [49] which correlates with poor outcome and metastases in breast cancer [50] and nasopharyngeal carcinoma [51] and tumour recurrence in hepatocellular carcinoma [11]. Finally the cancer upregulated drug resistant (CUDR) lncRNA, has demonstrated resistance to doxorubicin and etoposide, enhancing drug induced apoptosis following stable transfection [52]. These studies demonstrate the potential for lncRNAs to be used as prognostic and diagnostic markers in cancer.

Here, we identified a panel of lncRNAs that was capable of distinguishing benign mesothelium and MPM tissue with relatively high sensitivity (71.4%) and specificity (100%). In the cell lines studied, this panel had a sensitivity and specificity of 100%. In a study including 200 MPM cases, Klebe et. al. demonstrated that calretinin together with CD15 and BG8 were sensitive and specific enough to correctly classify all cases [53]. In comparison with other mesothelial markers including WT1, the lncRNA panel has higher specificity. Molecular panels such as the one reported here can provide great insights into the molecular changes occurring in the tumour and may turn out to be useful for screening purposesummary, we show that lncRNA expression is dysregulated in MPM compared with normal mesothelium, in what is to the best of our knowledge the first study of its kind. Our profiling studies revealed that AK130275, AK129685, EF177379, BX648695, NR_003584 and AF268386 are substantially up-regulated in MPM tumours compared to benign pleura, and are detectable in both FFPE and fresh-frozen MPM tissues. We also demonstrate that lncRNAs have potential prognostic and diagnostic utility. Further work is required to evaluate whether these lncRNAs are capable of differentiating malignant mesothelioma from lung cancer and benign asbestos-related diseases, and to reveal their specific functions in MPM pathogenesis.

## Supporting Information

File S1 **This file contains supplementary Tables S1, S2, S3 and S4.** Table S1: Validation of mRNA targets using the Oncomine Database. Table S2: Summary of over-enriched gene ontologies in MPM Table S3: Correlations between the top differentially expressed lncRNA and mRNAs Table S4: Over-enriched gene ontologies for co-expressed mRNA-lncRNA targets.
(XLSX)
